# Synergistic application of biochar and lime modulates rhizosphere microbiome, suppresses pathogens, and enhances disease resistance in sugarcane

**DOI:** 10.1186/s12866-025-04355-z

**Published:** 2025-10-02

**Authors:** Shakeel Ahmad, Mengrong Wang, Hongming Zhang, Yaowen Deng, Qingmin Liang, Bing He, Ronghui Wen

**Affiliations:** 1 State Key Laboratory for Conservation and Utilization of Subtropical Agro bioresources, College of Life Science and Technology, Guangxi University, Nanning, China; 2 Guangxi Key Laboratory of Agro-Environment and Agric-Products Safety, College of Agriculture, Guangxi University, Nanning, China; 3 Agricultural college, Shihezi University, Shihezi, Xinjiang 832003 P.R. China

**Keywords:** Soil acidification, Soil properties, Rhizosphere microbial communities, Pokkah boeng disease, Stress resistance

## Abstract

Soil acidification disrupts microbial communities and exacerbates plant disease susceptibility in agriculture. This study demonstrates that combined application of biochar (15 t ha⁻¹) and lime (1.5 t ha⁻¹) in acidic sugarcane fields significantly elevates soil pH (up to 46.7%) and organic carbon (SOC), driving profound restructuring of the rhizosphere microbiome. High-throughput sequencing revealed suppression of potentially pathogenic bacteria (*Solirubrobacteraceae*, *Acidothermus*, *Gaiella*) and fungi (*Curvularia*, *Scytalidium*, *Myrothecium*), alongside qPCR-confirmed reduction in *Fusarium* abundance. Concurrently, we observed significant enrichment of beneficial taxa including plant-growth-promoting bacteria (*Sinomonas*, *Bacillus*, *Brevibacillus*, *Micromonospora*, *Filomicrobium*, *OLB13*, *Arenimonas*) and fungi (*Trichoderma*, *Aspergillus*, *Talaromyces*, *Penicillium*, *Taifanglania*, *Redeckera*). FUNGuild analysis confirmed decreased relative abundance of fungal plant pathogen guilds, particularly under the combined treatment. Redundancy analysis identified pH, SOC, and acid phosphatase as key environmental drivers of microbial assembly. These microbiome shifts correlated with enhanced plant defense responses: increased phenylalanine ammonia-lyase and superoxide dismutase activities, reduced malondialdehyde content, and significantly suppressed pokkah boeng disease incidence (82.57% reduction) and severity. Our findings establish that biochar-lime synergy ameliorates soil constraints, functionally modulates the rhizosphere microbiome to suppress pathogens, and systemically enhances plant immunity, providing a sustainable strategy for managing soil-borne diseases in acidified agroecosystems.

**Graphical Abstract:**

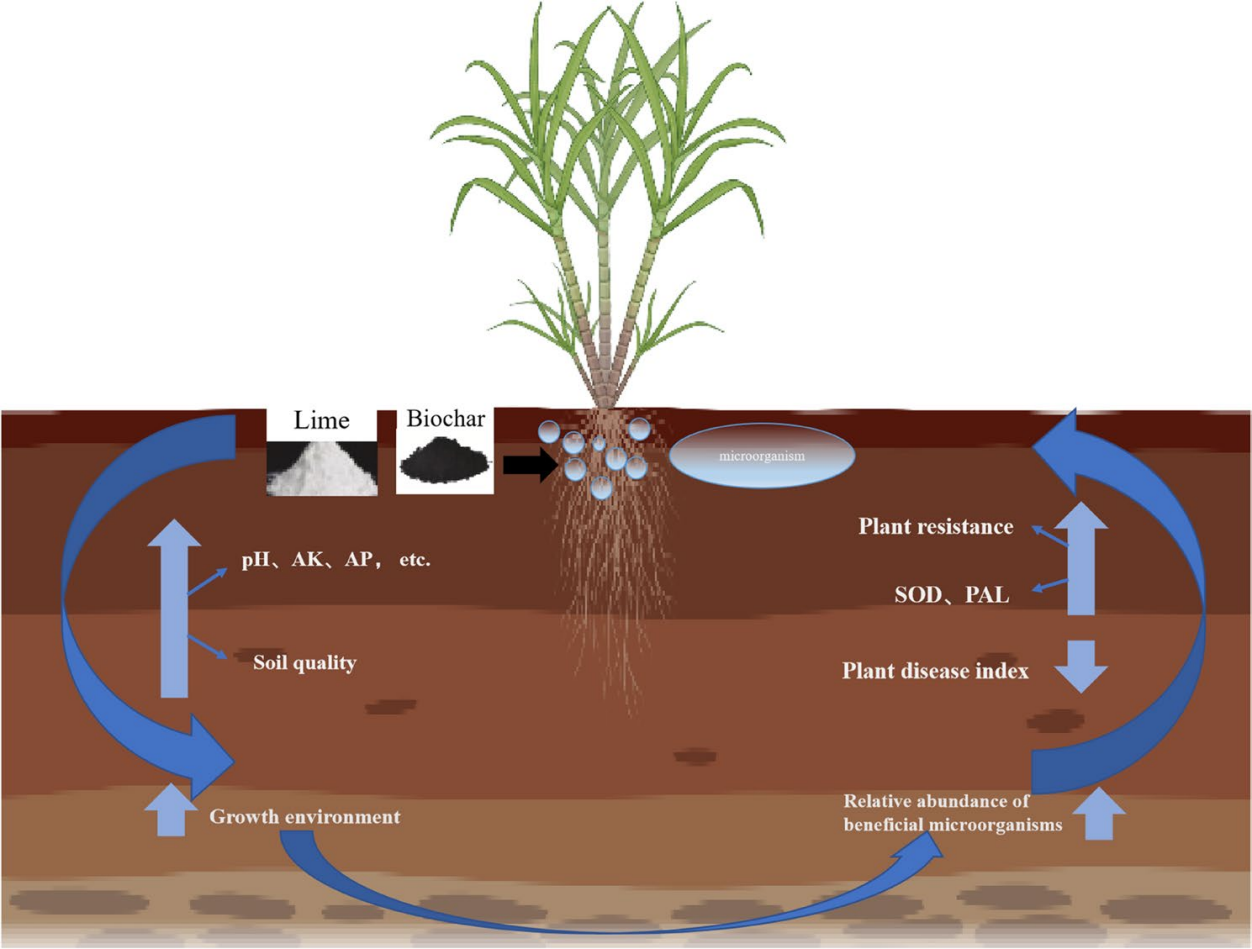

**Supplementary Information:**

The online version contains supplementary material available at 10.1186/s12866-025-04355-z.

## Introduction

Soil acidification remains a significant concern in China’s agriculture, with recent studies highlighting its ongoing impact. For instance, research indicates that from 1980 to 2023, the topsoil pH in Northeast China declined from 6.83 to 6.43, averaging a decrease of 0.0038 units per year [1]. These contrasting trends underscore the complex nature of soil pH dynamics across different regions in China. As the largest sugarcane-producing area in China, Guangxi has more than 70% red soil and lateritic red loam, having characteristics, such as soil acidity (pH 4.5-5.5), low soil nutrient content, poor fertilizer retention, and low phosphorus availability. Further, soil acidification reduces crop yields and Limits agricultural production. Most plants are suitable for growing with a value of 5.5།6.5 of soil pH [2]. However, since 2000, with the promotion of sensitive varieties such as ROC1, ROC10, ROC16, and ROC22, there has been a serious situation of frequent occurrence of pokkah boeng disease (PBD) in the entire sugarcane producing areas of China, and has caused significant economic losses [3]. The main influencing factors for the outbreak of PBD include large-scale planting of susceptible varieties, high temperature, humid climate, accumulation of pathogens, improper cultivation, and management measures [4]. Biological control offers a safer, environmentally friendly long-term solution to sugarcane PBD, but due to constraints, alternative sustainable strategies are required for effective disease management [5]. Soil acidification disrupts microbial activity and nutrient cycling, exacerbating the environmental impact of chemical control methods for sugarcane PBD, thus emphasizing the need for more sustainable, biologically-based disease management strategies [6]. Therefore, it is necessary to identify effective soil amendments to reduce soil acidification, which has become one of the critical conditions to maintain sustainable agricultural development and ensure food production.

The use of chalk or limestone (CaCO_3_), lime (CaO), or hydrated lime (Ca (OH)_2_) to increase soil pH and alleviate agricultural land acidification has been a common practice in agriculture for centuries [7]. The application of lime can change the chemical equilibrium of soil. Therefore, the mitigation of soil acidification also influences the chemical cycles and forms of C, N, and sulfur (S), which are fundamental to various agricultural and land use activities [8]. Moreover, biochar is usually alkaline improves the pH of acid soil [9, 10], and has a strong carbon fixation capacity [11, 12]. Other studies have shown that the application of biochar in acidic soil has increased its adsorption capacity for nutrients, and regulated sugarcane rhizosphere soil pH, NO_3_^−^-N, and potential nitrification [13].

The application of lime alone did not affect plant species diversity of plots were not fertilized or treated with sodium nitrate. However, when lime was combined with ammonium sulfate, it significantly increased plant species richness in these unfertilized plots [14]. In contrast, in the acidic sugarcane field, the addition of lime led to significant increase in the richness of soil microorganisms but reduced their diversity, as it decreased the evenness of spices distribution [15]. Additionally, lime has been shown to promotes the restoration of species richness with long-term N deposition in soil [16]. Further, changes in bacterial community composition influenced the soil nitrogen cycle [17], while changes in fungal proportion may impact the stability of soil aggregates, and the carbon content in fungal structure [18, 19]. Biochar provides unstable carbon and essential micro or macro nutrients to the microbial community [20, 21], also enhances microbial diversity by providing a habitat through its porosity and ability to adsorb soluble organic carbon, benefiting both bacteria and fungi [22, 23]. There are few studies on the use of lime to alleviate plant fungal diseases, but a large number of studies showed that biochar has changed the structure and abundance of plant rhizosphere microbial community, promoted plant growth, and improved plant resistance to pathogenic fungi [24]. The application of biochar from the raw material of citrus wood can control the gray mold (airborne) of tomato and pepper [25], and the application of biochar can also recover the rhizosphere injury caused by soil-borne pathogens *Fusarium oxysporum* f. sp. asperagi and *F. proliferatum* [26]. Recently, the prevention and control of PBD relies on chemical pesticides in the short term and disease resistance breeding in the long term, both of which have certain limitations. However, there are few studies on the improvement of acidified sugarcane fields, and the impact on PBD, after applying sole biochar and sole lime.

Lime and biochar individually demonstrate potential in ameliorating soil acidity and modifying microbial profiles, critical knowledge gaps persist regarding their synergistic impact on the functional restructuring of the sugarcane rhizosphere microbiome. Specifically, it remains unclear how combined application drives taxonomic and functional shifts in microbial communities, suppresses key soil-borne pathogens like *Fusarium*, and systemically primes plant immunity against PBD. To address this, we hypothesized that biochar-lime synergy would functionally remodel the rhizosphere microbiome by enriching beneficial taxa, suppressing pathogenic guilds, and activating plant defense pathways, ultimately mitigating PBD incidence more effectively than either amendment alone. This study therefore aims to: (1) Quantified how biochar-lime integration reshapes rhizosphere bacterial and fungal communities using high-throughput sequencing; (2) Assessed functional consequences via pathogen guild analysis and *Fusarium* suppression; and (3) Evaluated linkages between microbiome restructuring and induced plant resistance. Our findings establish a strategy for sustainable disease management in acidified agroecosystems.

## Materials and methods

### Experimental site and treatments

During 2021–2022, a field experiment was conducted at the Subtropical Agricultural Science Experimental Base in Fusui County, Chongzuo, Guangxi Province, China. The average air temperature is 18–27℃, and a total precipitation 1811.1 mm (22°29′15″north latitude, 107°45′21″east longitude). The basic soil properties were pH 4.77; available potassium (AK) 173.19 mg kg^−1^; available phosphorus (AP) 5.36 umol g^−1^; ammonium nitrogen (NO_3_^−^-N) 6.33 ug g^−1^; ammonium nitrogen (NH_4_^+^-N) 27.59 ug g^−1^; soil organic carbon (SOC) 24.7 g kg^−1^; SOM 42.58 g kg^−1^. While soil enzyme activities were soil β- Glucosidase (S-β-GC) 12.16U; soil catalase (S-CAT) 26.82 U; urease (S-UE) 490.55U; and soil acid phosphatase (S-ACP) 59613.81U. Treatments were arranged in a randomized complete block design with three replications in the field conditions. The biochar used in this study was produced locally from date palm fronds and leaves through slow pyrolysis at 500°C for 60 min, resulting in a fixed carbon content of 70.74%, a pH of 9.19, and a cation exchange capacity (CEC) of 68.05 cmol/kg. The biochar is primarily composed of palm fiber. The quicklime used contained ≥ 70% calcium oxide (CaO). The treatments included: G (GT42) and Z (ZZ9) varieties were considered as a control; GB, and ZB (Biochar, 15 t ha^−1^ B); GL, ZL (Lime, 1.5 t ha^−1^ L); GLB, and ZLB, combined application of 1.5 t ha^−1^ of Lime and 15 t ha^−1^ of biochar. Each treatment consist of three replication. Biochar and Lime were manually applied evenly to the soil surface following the sugarcane harvest. The land was then plowed and leveled using harrowing and rotary tillage with specialized agricultural machinery. Ditches were created at a row spacing of 1.2 m, and the compound fertilizer (NPK: 18-10-12) from Duodele Fertilizer Co., Ltd, China, was evenly distributed within the ditches at a rate of 0.45 tons ha^−1^, sugarcane double-bud segments were placed in the ditches at intervals of 0.3 m, after which the soil was covered to a depth of 5–10 cm using the machine. During the early stage of sugarcane jointing, white soil mounding was applied, at the same time, topdressing was performed with the same fertilizer at a rate of 1.2 tons ha^−1^. The experiment consisted of a total of 24 plots, each with an area of 24 m^2^ (4 m×6 m) having 2 m intervals in each replicate. Moreover, tillage, fertilization, and all other agronomic practices were carried out during the experimental period.

### Sample collection and processing

During 2021–2022, soil samples were collected from the rhizosphere of the sugarcane field at the maturity stage from three random plants. Plants were carefully dug out from the soil with the help of a shovel, and after gentle shaking of roots, tightly bound soil was collected. Rhizosphere soil samples were thoroughly mixed to obtain composite samples. All the collected soil samples were passed through a sieve of 2 mm mesh size and 2 g was stored at − 80°C for DNA extraction instead. The remaining soil samples were air-dried for subsequent analyses.

### Determination of soil chemical properties and enzyme activities

Each soil parameter was measured using standard laboratory procedures. Soil pH was determined by mixing 15 g of sieved soil with 30 mL of deionized water (1:2 ratio), followed by measurement using a glass electrode pH meter standardized with pH 7 and pH 4 buffer solutions. Soil organic carbon (SOC) was quantified using the dry combustion method, where soil samples were combusted at high temperatures, and the released CO₂ was measured to determine carbon content. Ammonium-nitrogen (NH₄⁺-N) was extracted from soil using a 1 mol·L⁻¹ KCl solution, followed by analysis using the indophenol blue colorimetric method to quantify ammonium concentration. Nitrate-nitrogen (NO₃⁻-N) was extracted with a 2 mol·L⁻¹ KCl solution, and nitrate concentrations were determined calorimetrically using the cadmium reduction method. Available Phosphorus (AP) was extracted using the Bray-1 method, which involves shaking soil with a solution of 0.025 N HCl and 0.03 N NH₄F, followed by colorimetric analysis of the phosphorus content. Available potassium (AK) was extracted using 1 N ammonium acetate at pH 7, and the potassium concentration in the extract was measured using flame photometry [27]. The determination of soil enzyme activities was performed as described by Zhang et al. [24]. The activities of soil acid phosphatase (S_ACP; BC0145), soil urease (S_UR; BC0125), soil catalase (S_CAT; BC0105), and soil β- Glucosidase (S_β-GC; BC0165) were determined using the soil enzyme kit from Solarbio Science & Technology Co., Ltd. (Beijing, China).

### Determination of phenylalanine ammonia-Lyase (PAL), superoxide dismutase (SOD) activities, and malondialdehyde (MDA) contents

Root samples were collected, immediately flash-frozen in liquid nitrogen, and stored at − 80°C for subsequent analysis. PAL activity was quantified using the CheKine™ micro phenylalanine ammonia lyase (PAL) activity assay kit (Catalog No. KTB1160) ABbkine, Wuhan, China, which employs a colorimetric method to measure the increase in absorbance at 290 nm due to the formation of trans-cinnamic acid. SOD activity was assessed using the SOD activity assay kit (BC1270) Solarbio, Beijing, China, utilizing the WST-1 method, where the reduction of WST-1 by superoxide anions produces a water-soluble formazan dye, and the decrease in absorbance at 450 nm is inversely proportional to SOD activity. MDA content was determined using the MDA content assay kit (BC0025) Solarbio, Beijing, China, based on the thiobarbituric acid-reactive substances (TBARS) assay, where MDA reacts with thiobarbituric acid to form a colored complex, and the absorbance is measured at 532 nm.

### Extraction of soil DNA and PCR amplification

Total soil DNA was extracted using the FastDNA™ SPIN Kit for Soil (MP Biomedicals, Solon, Ohio, USA) according to the manufacturer’s instructions. The extracted DNA concentration and purity were measured using the NanoDrop™ 2000 spectrophotometer (Thermo Fisher Scientific, Wilmington, Delaware, USA). The primer pairs 515 F/907R (515 F: 5′-GTGCCAGCMGCCGCGGT-3′; 907R: 5′-CCGTCAATTCMTTTRAGTTT-3′) were used to amplify the V4-V5 region of 16 S rRNA (Xu et al., 2022), and the primer pairs ITSF/ITSR (ITSF: 5′-CTTGGTCATTTAGAGGAAGTAA-3′; ITSR: 5′-GCTGCGTTCTTCATCGATGC-3′) were used to amplify the ITS I region of fungi. The PCR amplification was conducted using a 25-µL reaction mixture that contained 20 ng of DNA template, 0.5 µL of dNTP, 10 µL of KOD (kodakaraensis) polymerase buffer, 0.25 µL of Taq DNA polymerase (Takara Biotechnology, Dalian Co., Ltd., China), 5 µL of high GC enhancer, and 1.0 µL of each primer [28]. The first denaturation took place for 5 min at 98℃; then there were 25 rounds of 94℃ for 30 s, 52℃ for 30 s (annealing), 72℃ for 30 s (extension), and 72℃ for 10 min (final elongation). The PCR amplification was performed using a Bio-Rad S1000 thermocycler (Bio-Rad Laboratories, CA, United States). The Illumina MiSeq PE300 platform was used for DNA sequencing, and the subsequent analysis was carried out online on the Majorbio cloud platform (www.majorbio.com). To ensure sequence quality, low quality sequences with lengths of less than 200 bp and average quality score of less than 20 were filtered out. Operational taxonomic units (OTUs) clustering was performed on non-repetitive sequences (excluding single sequences) using the Ribosomal Database Project (RDP) Classifier with a 97% similarity threshold [29]. Non-redundant sequences, excluding singletons, were clustered into OTUs, with chimeric sequences removed during the clustering process. For taxonomic identification, bacterial OTUs were matched against the silva (https://www.arb-silva.de/) database comparison, and fungal OTUs were compared with the Unite (https://unite.ut.ee/) compare the database. All sequences have been deposited into the NCBI archive under Bio Project ID PRJNA1200795.

### Assessment of disease severity and absolute quantitative detection of Fusarium content

From June to July 2021, sugarcane growth reached the jointing stage, and PBD incidence was investigated. All leaves of each sugarcane plant were observed, and the total number of plants, number of affected plants, and the disease severity were recorded. The incidence level of PBD was based on Wang Zeping’s classification method [30]. The disease index of sugarcane pokkah boeng was determined by the method of Li et al. [31]. For quantification of *Fusarium* content in rhizosphere soil, total DNA was extracted the FastDNA™ SPIN Kit for Soil (MP Biomedical, Solon, Ohio, USA) according to the manufacturer’s instructions. The extracted DNA was amplified using synthetic specific primers ITS-Fu-F/R (5′-CGGATCTCTTGGTTCTGGCA-3′/5′-AGCTGTGCTTGAGGGTTGAA-3′) to target an approximately 418 bp region. The amplified product was cloned and sequenced, followed by homology alignment using DNAMAN software. The alignment result revealed 100% sequence homology with *Fusarium* (GenBank accession number: HQ674657.1), validating the specificity of both the primers and amplification product. Subsequently, this fragment was used to establish an absolute quantitative detection system for real-time fluorescence qPCR. The qPCR protocol was referenced from the method described by Xiu-yu et al. [32]. Each 20 µL qPCR reaction contained 10 µL of 2 × SYBR Green Master Mix (Tiangen Biotech, Beijing, China), 0.4 µL of each primer (10 µM), 2 µL of DNA template, and nuclease-free water to volume. Amplification was performed on a [ABI 7500 Fast Real-Time PCR System] with the following thermal cycling conditions: initial denaturation at 95°C for 3 min, followed by 40 cycles of 95°C for 10 s, 60°C for 30 s, and a melting curve analysis from 65 to 95°C. A 10-fold serial dilution of the cloned *Fusarium* ITS fragment was used to generate a standard curve R² >0.99 for absolute quantification. Reactions were run in triplicate. *Fusarium* content was expressed as DNA copy number per gram of soil.

### Statistical and bioinformatics analysis

One-way ANOVA analysis of variance was performed using Statistix 8.1 to analyze soil properties and enzyme activities. An LSD (Least Significant Difference) test at a p-value < 0.05 was used and figures were generated using GraphPad 8.0. Statistical analyses, including venn charts, community column charts, and community heatmap charts, were carried out using R language (version 3.3.1). Alpha diversity indices such as Shannon, Chao1, ACE and Simpson for microbial population among the treatments was assessed via Quantitative Insights Into Microbial Ecology (QIIME). Principal coordinates analysis (PCoA) based on the Bray-Curtis distance matrix was used to assess similarities and differences between groups and visualized with R. The Kruskal-Wallis H test with false discovery rate (FDR) correction was applied to adjust *p*-values for multiple comparisons. To study the ecological and functional classification of fungi, the FUNGuild (Fungi Functional Guild) V1.0 online platform was used. OTUs obtained from high-throughput sequencing were uploaded to FUNGuild, linking fungal species classification to functional guilds. Redundancy analysis (RDA) was performed to assess the relationship between soil properties and bacterial and fungal phyla, with soil nutrient-related attributes fitted in ordination plots using a 999-permutation test (*p*-values).

## Results

### Response of Biochar and lime on soil pH and SOC contents

The application of lime or biochar significantly increased the pH of the soil (*P* < 0.0001) in the rhizosphere soil of sugarcane (Table 1). The results showed that biochar amendments did not significantly affect soil pH. However, the treatment groups that applied lime such as GL, GLB, ZL, and ZLB were significantly higher than the control group (G, Z) (*P* < 0.0001). Compared with G, GLB, Z, ZL, and ZLB, the application of lime in the GT42 cultivar significantly increased soil pH by 46.7%, 5.61%, 49.1%, 23.5%, and 11.2%. Further, SOC content in the rhizosphere soil was significantly increased by 13.5% and 28.7% respectively in the GLB treatment plot compared with that of GL and Z (Table 1).

**Table 1:** Response of Biochar and lime amendments on soil pH, organic carbon, available P and K, and soil minerals nitrogen contents under different varieties of sugarcane

Treatments	pH	SOC (g kg^−1^)	AK (mg kg^−1^)	AP (mg kg^−1^)	NH_4_^+^-*N* (µg g^−1^)	NO_3_^−^-*N* (µg g^−1^)
G	4.6(0) ef	24.3(1) ab	152.3(4) b	4.9(0) d	3.6(0) a-d	2.9(1) b
GL	6.7(0) a	23.2(1) ab	104.7(1) e	4.9(0) cd	2.8(0) cd	6.0(1) ab
GB	4.6(0) fg	25.5(1) a	151.1(0) b	5.7(0) b	2.9(0) bcd	7.7(2) a
GLB	6.4(0) b	26.3(1) a	221.4(1) a	8.0(0) a	4.0(0) a	6.3(1) ab
Z	4.5(0) g	20.5(1) b	94.8(1) f	5.2(0) c	2.7(0) d	3.6(0) ab
ZL	5.5(0) d	25.2(1) a	118.8(1) d	4.1(0) e	3.4(0) a-d	7.1(2) ab
ZB	4.7(0) e	24.8(0) ab	109.7(2) e	4.8(0) d	3.8(0) ab	5.1(1) ab
ZLB	6.1(0) c	24.9(0.1) ab	127.3(2) c	5.6(0) b	3.7(0) abc	5.1(1) ab

*G* No application to the GT42 variety, *GL* Application of 1.5 t ha⁻¹ lime to GT42, *GB* Application of 15 t ha⁻¹ biochar to GT42, *GLB* Combined application of 1.5 t ha⁻¹ Lime and 15 t ha⁻¹ biochar to GT42, *Z* No application to the ZZ9 variety, *ZL* Application of 1.5 t ha⁻¹ lime to ZZ9, *ZB* Application of 15 t ha⁻¹ biochar to ZZ9, *ZLB* Combined application of 1.5 t ha⁻¹ Lime and 15 t ha⁻¹ biochar to ZZ9. Numbers followed by different lowercase letters in a column indicate significant differences (*P* ≤ 0.05). Values within each column show statistically significant differences among each other

### Response of Biochar and lime on soil available K, P, and mineral N contents

The available potassium (AK) content was significantly affected by different treatments (*P* < 0.0001), while ZL significantly reduced the AK content by 31.2% compared with G (control). Further, 73.9% of available K content was significantly increased in the GLB treatment compared with that of ZLB (Table 1). The treatment group GLB significantly improved soil AP content by 63.8%, 62.6%, 41.7%, 53.9%, 97.9%, 65.8%, and 43.0% compared with the treatment groups such as G, GL, GB, Z, ZL, ZB, and ZLB respectively. Moreover, in the rhizosphere soil of the GT42 variety, only GLB treatment was 11.79% higher NH_4_^+^-N than the control (G), and in the ZZ9 variety, the treatments (ZL, ZB, and ZLB) were 27.60%, 43.53%, and 37.16% higher than the control (Z). Further, GLB treatment significantly increased the content of NH_4_^+^།N by 43.3%, 39.1%, and 51.0% compared with GL, GB, and Z. While, compared with G and Z, the application of lime or biochar (GL, GB, GLB, ZL, ZB, and ZLB) increased the soil NO_3_^−^-N content by 108.92%, 167.86%, 117.86%, 97.14%, 45.71%, 40.00% at the maturity of sugarcane of two different varieties of sugarcane (Table 1).

### Effects of Biochar and lime on soil enzyme activities

Treatments (GB, ZB) significantly reduced the S-UE activity by 15.28%, 86.04% compared with control (G, Z) in the rhizosphere soil of sugarcane, while, GLB significantly improved S-UE activity by 28.1%, 18.6%, and 29.7% compared with GB, Z, and ZB, respectively (Table 2). Further, GLB and ZLB consistently reported higher S-UE activity than control (G, Z). While, GL, GLB, ZL, and ZLB significantly reduced S-ACP activity by 50.53%, 58.39%, 50.45%, and 62.70% compared with their respective control (G, Z) (Table 2). The S-CAT activity increased by 42.27%, 45.38%, 12.77%, and 18.38% with the treatments GL, GLB, ZL, and ZLB, respectively, compared with control (G, and Z). Additionally, the treatments GB and ZB significantly increased S-CAT activity by 8.89% and 3.89% (*P* < 0.005) compared with control (G and Z). In contrast, the treatments GL, GB, GLB, ZL, ZB, and ZLB decreased the activity of β-glucosidase by 26.58%, 29.50%, 54.17%, 13.14%, 42.31%, and 4.78%, respectively, compared with control (G and Z).

**Table 2:** Response of Biochar and lime amendments on soil urease, acid phosphatase, β-glucosidase, and catalase activities (U g^−1^) under different varieties of sugarcane

Treatments	S-UE	S-ACP	S-β-SC	S-CAT
G	515.4(1) ab	59,768(619) a	14.1(1) a	22.7(1) f
GL	558.1(17) a	29,568(125) c	10.3(1) b	32.3(0) a
GB	436.7(6) c	57,671(130) a	9.9(1) bc	24.7 ± 0.14e
GLB	559.3(18) a	24,868(381) d	6.5(0) d	33.0(1) a
Z	471.7(8) bc	59,460(858) a	13.2(1) a	25.5(1) de
ZL	552.6(3) a	29,461(683) c	11.5(1) ab	28.7(0) c
ZB	431.1(14) c	46,327(181) b	7.6(1) cd	26.5(1) d
ZLB	519.9(19) ab	22,178(346) d	12.6(1) ab	30.2(0) b

*G *No application to GT42 variety, *GL* Application of 1.5 t ha^−1^ lime to GT42, *GB* Application of 15 t ha^−1^ biochar to GT42, *GLB* Combined application of 1.5 t ha^−1^ Lime and 15 t ha^−1^ biochar to GT42, *Z* No application to ZZ9 variety, *ZL* Application of 1.5 t ha^−1^ lime to ZZ9, *ZB* Application of 15 t ha^−1^ of biochar to ZZ9, *ZLB* Combined application of 1.5 t ha^−1^ Lime and 15 t ha^−1^ of biochar to ZZ9. Numbers followed by different lowercase letters in a column indicate significant differences (*P* ≤ 0.05). Values within each column show statistically significant differences among each other

### Effects of Biochar and lime on rhizosphere microbial diversity and community composition

After analyzing the bacterial diversity data of 72 samples, a total of optimized sequences 2,959,922, and 114,565,458 bases were obtained, with an average sequence length of 376 bp, and a community coverage of about 98%. Similarly, after analyzing the fungal diversity data of 72 samples, 3,704,554 optimized sequences, and 878,955,206 bases were obtained, with an average sequence length of 237 bp. The community diversity was 98% for both bacteria and fungus.

Venn diagram illustrated the number of unique and shared OTUs in the rhizosphere samples (Figure S1). In the bacterial group, the common OTUs number shared by all samples was 1421 and the unique OTUs numbers were 1, 0, 2, 16, 2, 0, 0, and 4 respectively. The application of lime and biochar increased the number of soil OTUs (Figure S1). The addition of Lime and biochar has been shown to influence the composition and structure of soil fungal communities. While, the total OTUs number shared by all samples was 436 and the unique OTUs number were 9, 3, 9, 6, 3, 8, 13, and 11, respectively (Figure S1).

**Fig. 1:**
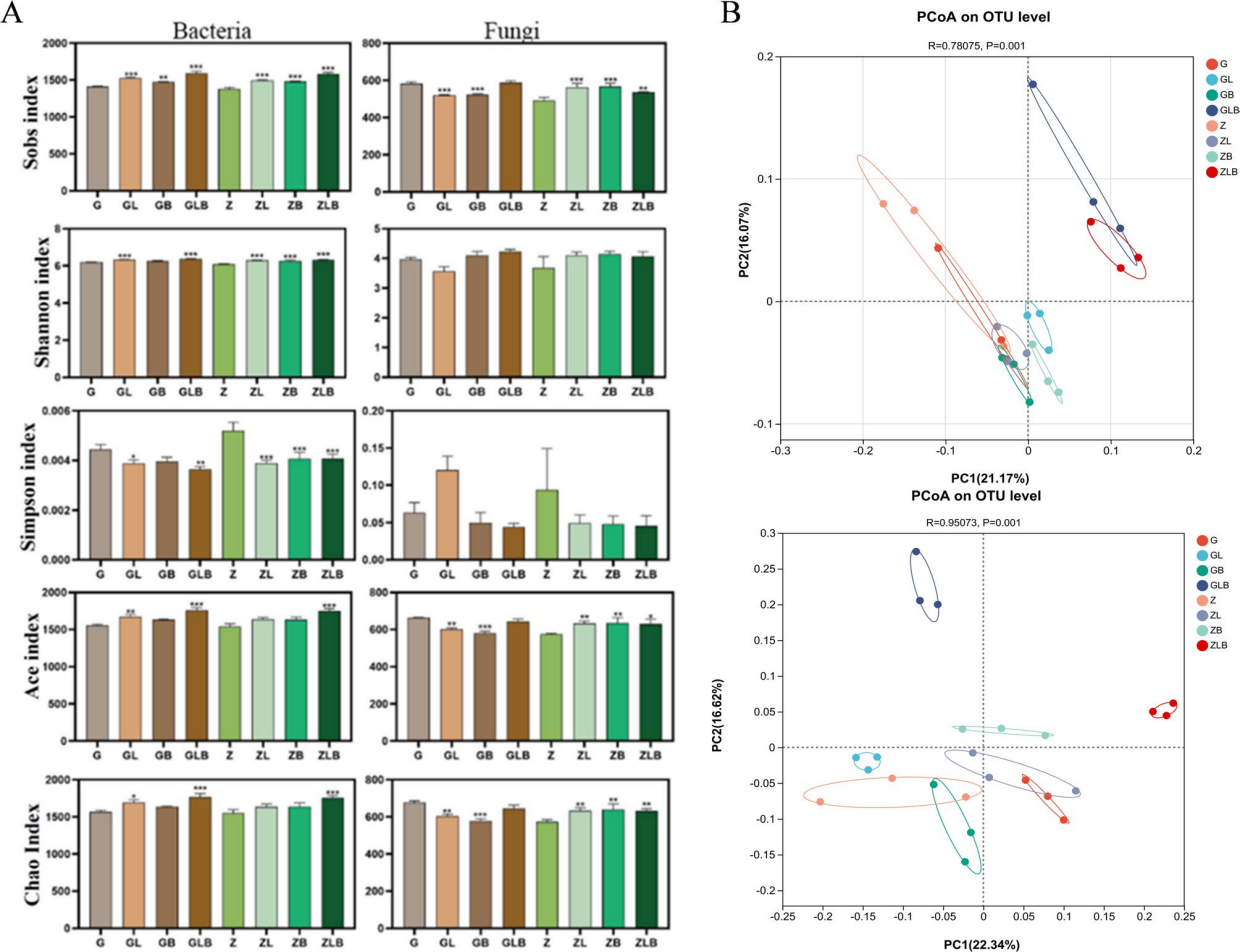
Alpha diversity analysis using the Shannon, Ace, Choa 1, and Simpson indices. The * indicate the significance level (*p* < 0.05) of the test at the top of each column (**A**) and PCoA analysis for Beta Diversity, using Bray circuit distance, and Adonis test (*p* < 0.05) (**B**) of sugarcane rhizosphere microbiome in various treatments

The treatments GL, GB, GLB, ZL, ZB, and ZLB significantly improved the soil bacterial community richness and diversity compared with the control (G, Z) (*P* < 0.001). Treatments GLB and ZLB showed a significant increase in soil bacterial community richness and diversity (Fig. 1A). Through comparative analysis of soil fungal richness and diversity, the treatments GLB, ZL, ZB, and ZLB significantly improved the richness and diversity of soil fungi compared with the control (G, and Z). Principal coordinate analysis (PCoA) of soil bacterial and fungal community structure (Fig. 1B), indicated that the treatment groups with the same treatment are more likely to cluster together.

**Fig. 2:**
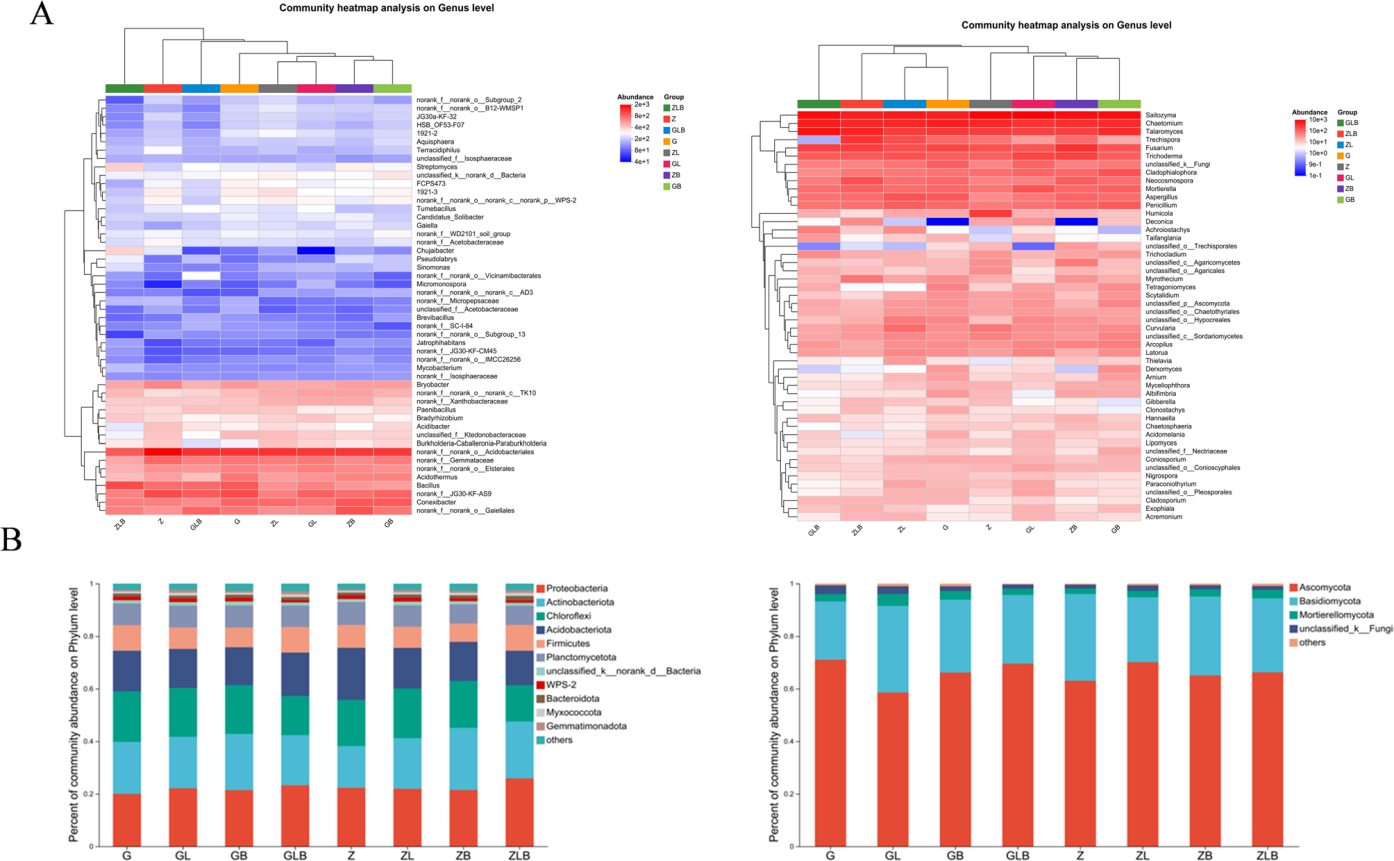
Heatmap of soil bacterial and fungal community composition at genus level. Variation in color represent the changes in the community composition in positive or negative way, and the various color on the right represent different treatments and at the top of the each histogram represent the different groups clustered together (**A**), Bacterial and fungal community relative abundance (**B**) histogram (phylum level) of sugarcane rhizosphere. The different color represents the various species and their relative abundance

Analyze the community composition between the control and the treatment group at the subordinate level based on the top 50 species with total abundance. From the perspective of bacterial community composition (Fig. 2A), the treated groups GL and ZL, GB and ZB, GLB and ZLB have similar compositions and can separated from the control (G and Z). From the perspective of fungal community composition (Fig. 2A), treatment GB and ZB were relatively similar and separate from other treatments.

Compared with the control group, the application of biochar with lime showed a difference in soil bacterial community composition and its relative abundance from the data of bacteria phylum level (Fig. 2B). During the maturity stage the *Proteobacteria* (19.98%~25.88%), *Actinobacteria* (15.88%~23.63%), *Chloroflexi* (13.85%~19.21%), *Acidobacteriota* (13.06%~19.82%), *Firmicutes* (7.02%~9.86%) were observed respectively. While analyzing the differences in the composition and relative abundance of soil fungal communities at the fungal phylum level (Fig. 2B). During the maturity stage, Ascomycota (58.59%~71.06%), Basidiomycota (22.15%~33.00%), Mortierellomycota (2.15%~4.49%), unclassified_ K__Fungi (1.39%~3.33%) were observed respectively.

### Analysis of species differences in sugarcane rhizosphere microorganisms

**Fig. 3:**
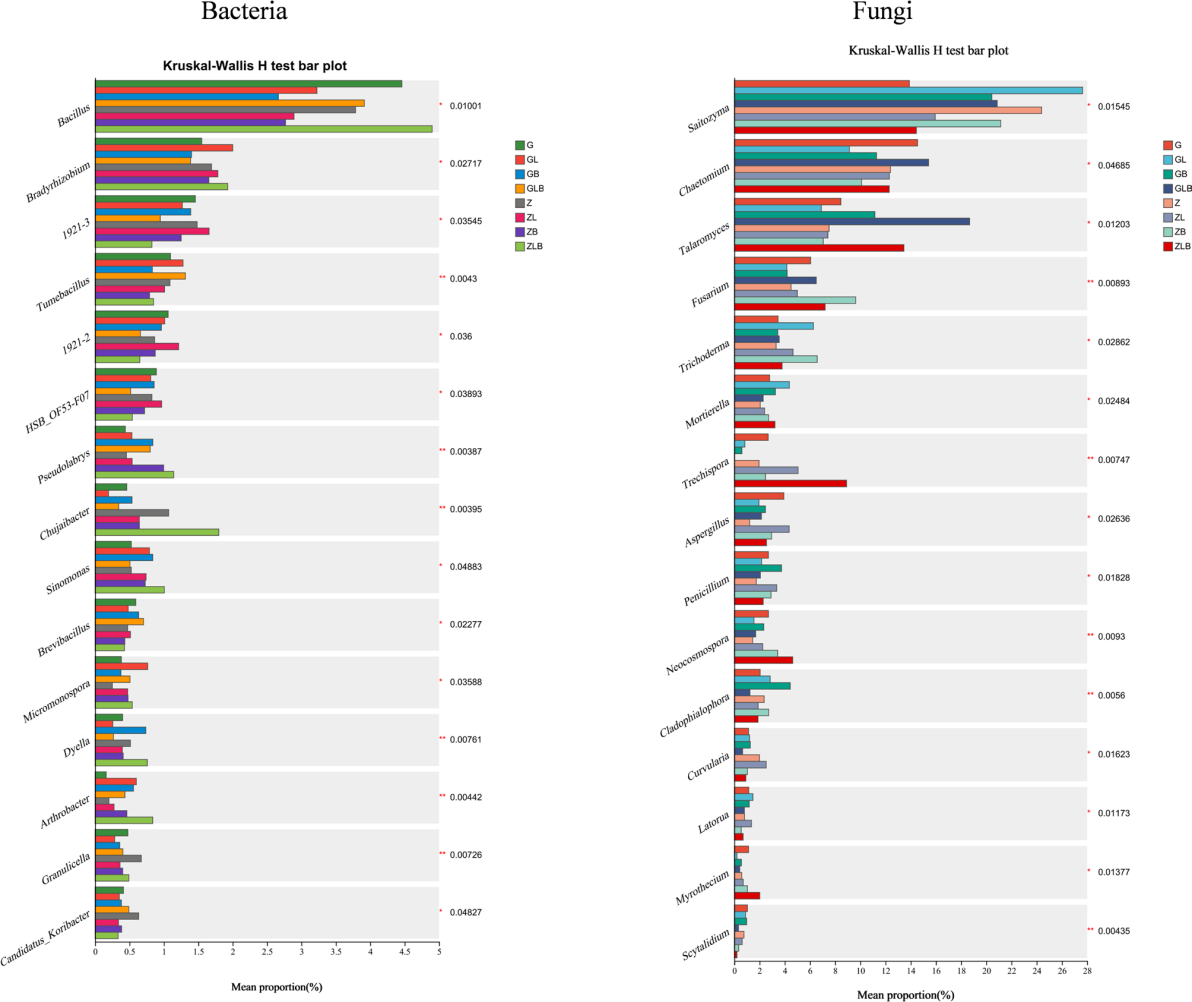
Beta diversity of bacterial and fungal community of sugarcane rhizosphere using Kruskal-Wallis H test. The various colors show different treatments and * show the significance level (*P* < 0.05). Note: Data in the figure was corrected for multiple tests using fdr, while the Post-hoc test used Tukey kramer with a value of 0.95.

To understand the significant changes in species at the subordinate level, the Kruskal Wallis rank sum test was used to obtain the species with significant differences between groups. Figure 3 showed that the application of lime or biochar at the maturity stage of sugarcane significantly increased the relative abundance of *Pseudolabs*,* Sinomonas*,* Micromonospora*,* and Arthrobacter* (*P* < 0.05), and significantly decreased the relative abundance of Granulicella (*P* < 0.05). Only *OLB13*,* Arenimonas*,* Rhodopirellula*, and *Glycomyces* were significantly present in the combined lime and biochar treatment group (*P* < 0.05). While, the application of lime or biochar at maturity of sugarcane significantly increased the relative abundance of *Trichoderma* (*P* < 0.05), and decreased *Scytalidium* abundance (*P* < 0.05). *Oxyporus* was significantly present in the treatment group that applied combined lime and biochar (*P* < 0.05).

### Relationship between soil functionalities with rhizosphere microorganisms

**Fig. 4:**
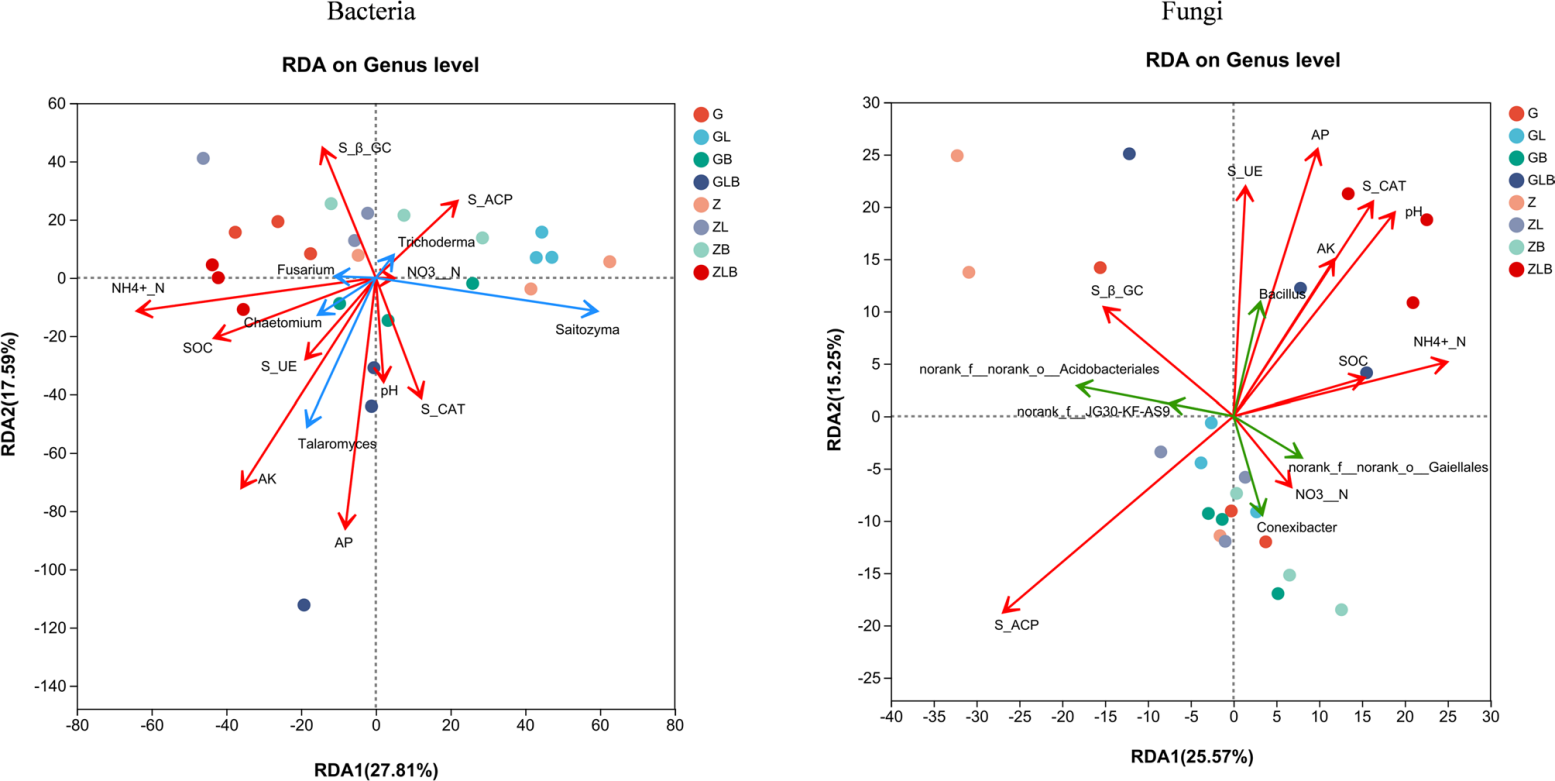
db-RDA analysis between soil properties and sugarcane rhizosphere microbial communities

Redundancy analysis (RDA) was performed to assess the relationship between soil biochemical properties and microbial community composition in the rhizosphere (Fig. 4). RDA1 showed variation on the X-axis and Y-axis by 25.57% and 15.25% for the bacterial community, while 27.81% and 17.59% for the fungal community at the maturity stage of sugarcane. The bacterial community showed that *Conexibacter* was significantly positively correlated with S- β-GC and S-ACP, while *Acidobacteries* significantly positively correlated with S-β-GC. *Bacillus* was positively correlated with all soil properties except S-β-GC at the maturity stage of sugarcane. Similarly, fungal communities showed that *Fusarium* significantly positively correlated with NH_4_^+^-N, *Chaetomium* with SOC and S-UE, *Taiaromyces* with AP and AK, while *Trichodema* significantly positively correlated with S-ACP at maturity of sugarcane.

### Functional prediction analysis of sugarcane rhizosphere fungal community

**Fig. 5:**
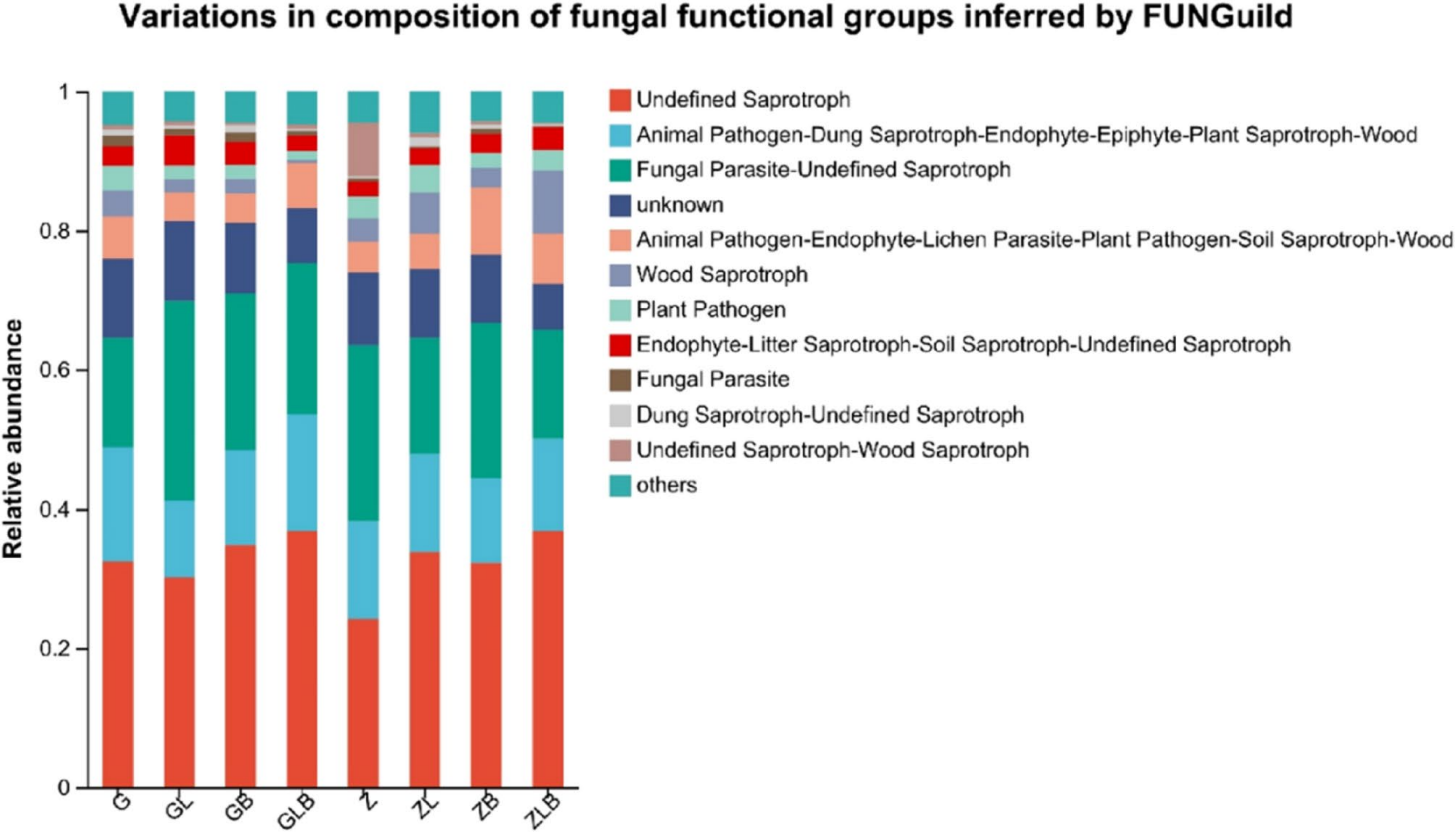
FUNGuild functional classification statistical bar chart of fungi

FUNGuild (Fungi Functional Guild) can classify and analyze fungal communities through micro ecological guides. Fungal OTUs are categorized into specific nutrient groups, which are further subdivided into specific ecological groups. Undefined Saprotroph, Fungal parasites, Animal pathogens, and unknown fungi account for approximately 80% of the tested OTUs (Fig. 5). The application of lime or biochar can generally reduce the relative abundance of plant pathogens in the fungal community, but the relative abundance of ZL plant pathogens was higher than control group (Z). Combining biochar with lime was always lower than the control group, and the relative abundance of the pathogen community was the lowest in most cases in this group.

### Effect of Biochar and lime on PAL, SOD activities, and MDA content of sugarcane roots

**Fig. 6:**
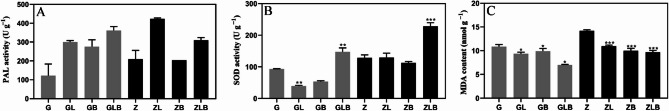
Effect of biochar and lime on Phenylalanine ammonia lyase (PAL), superoxide dismutase (SOD) activities and malondialdehyde content. G: no application to GT42 variety; GL: application of 1.5 t ha^-1^ lime to GT42; GB: application of 15 t ha^-1^ biochar to GT42; GLB: combined application of 1.5 t ha^-1^ Lime and 15 t ha^-1^ biochar to GT42; Z: no application to ZZ9 variety; ZL: application of 1.5 t ha^-1^ lime to ZZ9; ZB: application of 15 t ha^-1^ of biochar to ZZ9; ZLB: combined application of 1.5 t ha^-1^ Lime and 15 t ha^-1^ of biochar to ZZ9. * The LSD-test showed significant differences (*, *p* < 0.05;* *, *p* < 0.01; * *, *p* < 0.001). Values within each column showed statistical significantly difference among each other

Application of biochar and lime (GLB) significantly increased the PAL activity by 196.17% compared to G in GT42, whereas in ZZ9, the highest level of PAL activity was observed in ZL by 101.90% compared with Z, ZB and ZLB (Fig. 6A). SOD activity was significantly higher in Z compared with G. Results showed that the combined application of biochar and Lime, specifically treated with GLB and ZLB, significantly increased SOD activity by 57.29% and 77.34% compared with control and other treatments (Fig. 6B). Furthermore, biochar and Lime significantly decreased the MDA accumulation compared with G and Z. The results demonstrated that treatment GLB significantly reduced the MDA contents by 35.64% compared with G and other treatments; whereas, ZLB decreased the MDA content by 31.82% followed by ZL and ZB compared with Z (Fig. 6C).

### Effects of biochar and lime on disease index and absolute content of Fusarium in sugarcane rhizosphere

**Fig. 7:**
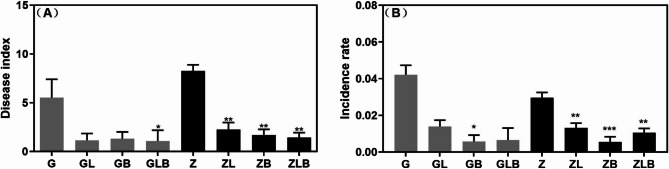
Response biochar and lime amendments on the disease index **A** and incidence rate **B** of pokkah boeng disease (PBD) in sugarcane rhizosphere of two different varieties

The application of lime and biochar significantly reduced the disease index and incidence rate of PBD (*P* < 0.005; Fig. 7), and the disease index of GT42 was lower than that of ZZ9. The treatment group (GLB, ZLB) had the lowest disease index; however, the disease index decreased by 82.57% by the susceptible variety (ZZ9). According to the amplification curve provided by the standard concentration gradient and reaction cycle threshold, the standard curve was plotted using GraphPad 9.0, with a correlation coefficient of R^2^ = 0.9951 and a slope of − 3.983. Subsequently, qPCR was used to explore the DNA extracted from samples of each treatment group at different periods and the Cp values obtained were brought into the absolute quantitative standard curve for calculation. The results are shown in the supplementary file (Figure S2). The content of *Fusarium* in the rhizosphere of sugarcane at the maturity stage was significantly lower in all treatment groups than in the control group (*P* < 0.001). However, the content of *Fusarium* in the treatment group (GLB and ZLB) was the lowest.

## Discussion

### Response of Biochar and lime on soil functionalities

In this study, we hypothesized that the combined application of biochar and lime would significantly improve soil quality and nutrient availability compared to individual amendments. Soil chemical properties, such as pH, and enzyme activities are key indicators of soil health [33]. Lime application resulted in a significant increase in soil pH, aligning with well-established practices that use lime to neutralize acidity and improve plant growth conditions [34]. Biochar has also been reported to increase pH, but its effectiveness varies depending on factors like soil type and biochar composition [35, 36]. Our findings support the notion that specific soil characteristics and amendment properties need to be considered when modifying pH.

Further, applied biochar extracted from oil palm husk (*Elaeis guineensis*), *Theobroma cacao* L., and rice husk (*Oryza sativa* L.) to 31 kinds of acid soils, and observed that biochar from cocoa husk increased the soil pH from 4.7 to 5 [37]. The combined application of lime and biochar significantly enhanced the soil organic carbon (SOC) content at sugarcane maturity. This increase is likely due to lime’s ability to reduce soil acidity and indirectly enhance carbon accumulation [38]. Numerous studies have showed that biochar has well-documented effects on improving soil structure and stabilizing carbon content [39]. The integration of both amendments appears to offer sustained benefits to SOC compared to individual treatments.

In terms of nutrient availability biochar significantly increased available K, whereas lime decreased it, possibly due to lime influence on the soil’s potassium (K) adsorption capacity [40]. The combined amendments, however, increased both AK and AP. Lime may increase AP by mineralizing soil organic phosphorus and improving aluminum toxicity, while biochar influences phosphorus solubility by altering soil pH and promoting phosphorus-solubilizing microbes [41]. The combine treatment also increased NO_3_^−^-N content, suggesting improved nitrogen availability, but the NH_4_^+^-N content varied with different planting varieties, indicating that the amendments effect on nitrogen pool may depend on the crops type. The application of both lime and biochar promoted nitrification and reduced nitrate leaching, improving nitrogen retention and availability for crops [42].

Enzyme activities, such as S-UE and S-CAT, increased with lime and the combined treatments, likely due to the positive correlation between soil pH and microbial activity [43]. However, acid phosphatase (S-ACP) activity decreased with lime application, which is consistent with its known inverse relationship with pH [44]. Interestingly, the combined treatment of biochar and lime reduced beta-glucosidase (S-\u03b2-GC) activity, possibly due to a reduction in the availability of carbon-rich substrates for microbial use [44].

### Microbial community structure and composition

The combined application of biochar and lime significantly enhanced overall microbial diversity and richness, likely due to improved nutrient availability and soil structure. However, this increase did not correspond to substantial changes in fungal community diversity during the early stages of the experiment. One possible explanation is the limited mobility of fungal hyphae, which tend to form stable associations with soil particles, thereby reducing their ability to rapidly respond to environmental changes. Moreover, fungal communities are ecologically more stable and slower to shift compared to bacterial communities, owing to their longer life cycles and more complex structural networks [22]. This ecological resilience may account for the delayed response observed in fungal diversity, despite the overall increase in microbial diversity. Thus, there is little change in fungal diversity in the early growth stages. Analysis of the microbial community histogram revealed that the application of biochar and lime significantly altered the composition of the soil microbial community. This effect is likely due to the amendments’ influence on soil environmental factors, such as pH, nutrient availability, and organic matter content, which are known to shape microbial community structures. Microbial community analysis revealed that biochar and lime significantly altered microbial composition. At the phylum level, the main bacterial groups enriched by the combined treatment included *Proteobacteria*, *Actinobacteriota*, *Chloroflexi*, *Acidobacteriota*, and *Firmicutes*. *Proteobacteria*, which are known to thrive in nutrient-rich environments, increased significantly under the combined treatment, reflecting improved soil fertility [45]. The fungal groups enriched by the combined treatment included *Ascomycota*, *Basidiomycota*, and *Mortierellomycota*, with *Mortierellomycota* particularly standing out for its beneficial properties, such as pathogen antagonism and production of nutritional compounds [46].

At the genus level, notable enrichments included *Sinomonas*, *Bacillus*, *Brevibacillus*, and *Micromonospora*. These genera play important roles in promoting plant growth, nitrogen cycling, disease suppression, and bioremediation [47]. Conversely, in the control group, pathogenic fungi such as *Curvularia*, *Albifimbria*, and Myrothecium were more abundant. Bacillus, known for its biocontrol properties, showed a negative correlation with the presence of *Fusarium*, suggesting its potential in managing soil borne diseases [48, 49]. The results highlight that the combined application of biochar and lime can significantly alter the microbial landscape in favor of beneficial microbes that enhance soil health and plant resilience, while suppressing pathogenic organisms.

### Ecological and soil health implications

Soil properties particularly pH, significantly influence microbial community composition and abundance, which can explain the observed differences in microbial populations [50]. The application of biochar and lime changed the key soil factors such as pH, SOC, and AP, which in turn influenced microbial suppression of acidophilic taxa like *Acidobacteriota* suggest that the amendments created a more favorable environment for microbial diversity [51]. Changes in soil pH also impacted the adsorption and availability of phosphorus, which is critical for microbial growth [52]. The combined amendments enriched key phosphate-solubilizing genera such as Bacillus, Penicillium, and Aspergillus, which are important for nutrient cycling and soil health [28, 53]. The increased availability of phosphorus, coupled with enhanced microbial activity, likely contributed to better plant nutrient uptake and overall soil fertility. Additionally, the observed negative correlation between Bacillus and *Fusarium* suggests that the treatment could reduce the incidence of root rot diseases, supporting the idea that soil health improvements can mitigate plant stress and disease [47]. Our results underscore the importance of microbial community management for soil health. The enriched microbial diversity under the combined biochar and lime treatments reflects improved nutrient cycling, disease suppression, and overall soil quality. This supports the idea that improving microbial communities through soil amendments can lead to more sustainable agricultural practices.

### Disease suppression and plant stress resistance

Biochar and lime would enhance plant immune responses, improving stress resistance and reducing disease incidence. Our results showed that the combined amendments increased PAL and SOD activities while reducing MDA content, suggesting enhanced plant defense mechanisms and reduced oxidative stress [54]. GT42 variety showed higher PAL activity, indicating its greater resistance to diseases compared to ZZ9, which aligns with previous findings on resistant varieties [55]. The combined amendments also significantly reduced the disease index of sugarcane PBD, a major fungal disease caused by *Fusarium*. This reduction was associated with lower *Fusarium* populations in the rhizosphere, as confirmed by qPCR analysis. The amendments likely suppressed both airborne and soil borne pathogens by creating a more favorable microbial environment and improving soil conditions [47, 56]. These results suggest that improving soil microbial communities through biochar and lime amendments can effectively reduce disease incidence and enhance plant resilience, offering a sustainable solution for managing soil borne pathogens. Remarkably, the combined application of biochar and lime (GLB and ZLB treatments) consistently resulted in higher yields compared to their individual applications, with the ZLB treatment achieving the highest yield (Table S1). Overall, results indicate that the combined application of biochar and lime significantly improved soil functionality, microbial diversity, and plant health. The amendments enhanced nutrient availability, supported beneficial microbial communities, and suppressed pathogens, contributing to better soil health and disease management. Additionally, we acknowledge that the ecological cost of biochar application is currently high, and its use in sugarcane cultivation is not yet widespread. Our findings underscore the potential of integrated soil management practices that improve both soil microbial ecology and crop resilience. Future research should focus on optimizing application rates and exploring the long-term effects of these amendments on soil health and crop productivity.

## Conclusions

The combined application of biochar and lime significantly increased soil pH, AK content, and AP, while enhancing S-UE and S-CAT activities. NO_3_^−^-N content increased, whereas, NH_4_^+^-N responses varied by sugarcane varieties. Microbial richness, particularly fungal diversity at the maturity stage was significantly improved. Beneficial taxa such as *Proteobacteria* and *Mortierellomycota* were enriched, while potentially pathogenic genera (*Solirubrobacteriaceae*,* Acidothermus*,* GaiellaCurvularia*,* Albifimbria*,* Trichocladium*,* and Myrothecium*) were reduced. Moreover, the increased activities of PAL and SOD, along with reduced MDA content in sugarcane roots, suggest enhanced stress resistance. Although SOC content increased, this short-term change should be interpreted continuously, as SOC accumulation is influenced by seasonal dynamics and agronomic management, and requires long-term assessment. Overall, biochar and lime application rapidly improves soil nutrients status, microbial diversity, plant physiological responses, and disease resistance in sugarcane.

## Supplementary Information

Supplementary Material 1.

## Data Availability

All sequences have been deposited into the NCBI archive under Bio Project ID PRJNA1200795 and supplemental file.
