# Supplementary material for: Synergistic application of biochar and lime modulates rhizosphere microbiome, suppresses pathogens, and enhances disease resistance in sugarcane
Source: BMC Microbiol. 2025 Oct 2;25:622. doi: 10.1186/s12866-025-04355-z (PMC12492682; doi:10.1186/s12866-025-04355-z)
Supplement: Supplementary file 1 — Supplementary Material 1. [file 12866_2025_4355_MOESM1_ESM.docx]

Table 1 Response of biochar and lime amendments on sugarcane varieties in acidic soil conditions

| Guitang-42 (G) | | Zhongzhe 9 (Z) | |
| --- | --- | --- | --- |
| Treatments | Yield ha⁻¹ | Treatments | Yield ha⁻¹ |
| G | 78.45±4.38 | Z | 95.28±4.86 |
| GL | 82.64±4.98 | ZL | 99.63±6.87 |
| GB | 84.38±6.15 | ZB | 105.25±6.38 |
| GLB | 89.76±5.41 | ZLB | 112.76±5.53 |

Note: G = no application to the GT42 variety; GL = application of 1.5 t ha⁻¹ lime to GT42; GB = application of 15 t ha⁻¹ biochar to GT42; GLB = combined application of 1.5 t ha⁻¹ lime and 15 t ha⁻¹ biochar to GT42; Z = no application to the ZZ9 variety; ZL = application of 1.5 t ha⁻¹ lime to ZZ9; ZB = application of 15 t ha⁻¹ biochar to ZZ9; ZLB = combined application of 1.5 t ha⁻¹ lime and 15 t ha⁻¹ biochar to ZZ9.

**(A) (B)**


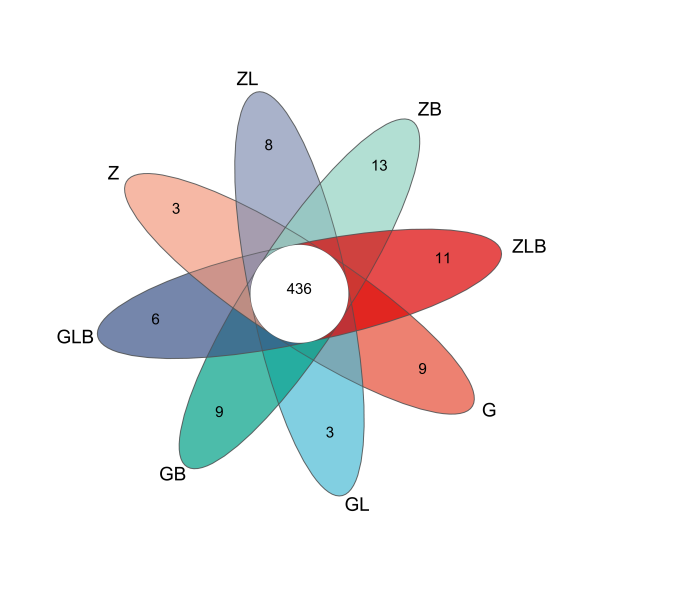

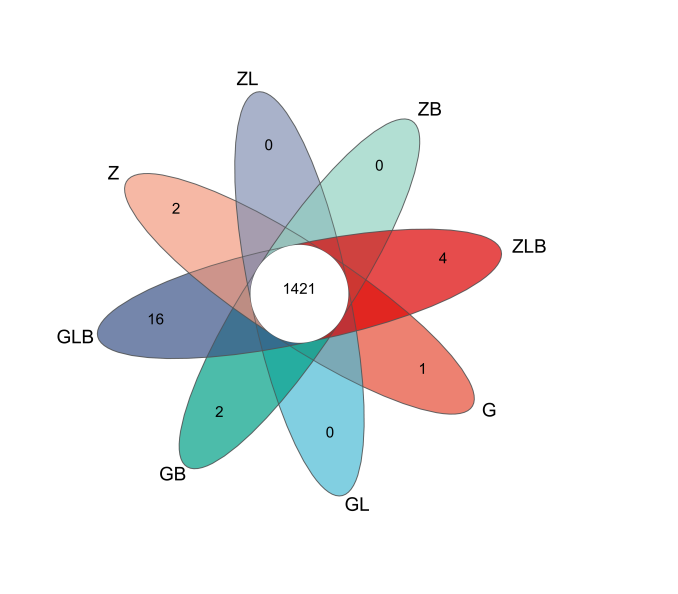


Figure S1 Venn diagram illustrating the number of shared and unique OTUs in the rhizosphere bacterial (A) and fungal (B) communities.

**(A)**


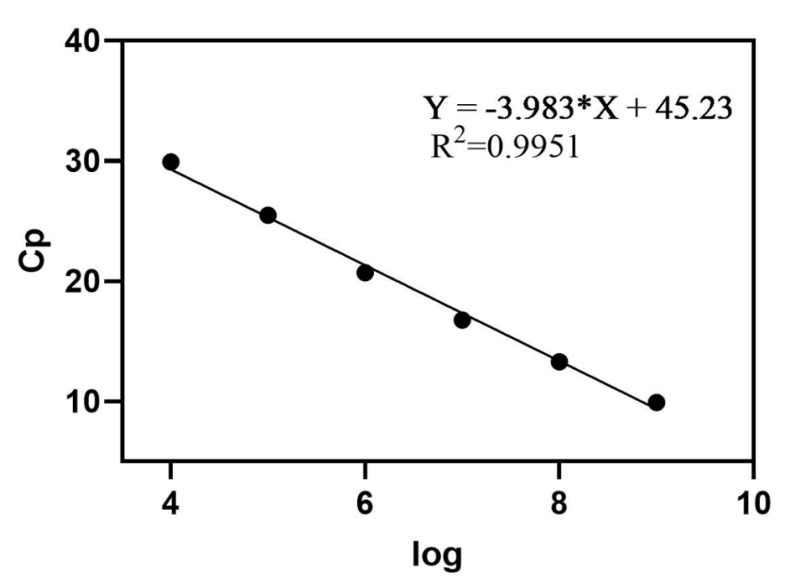


**(B)**


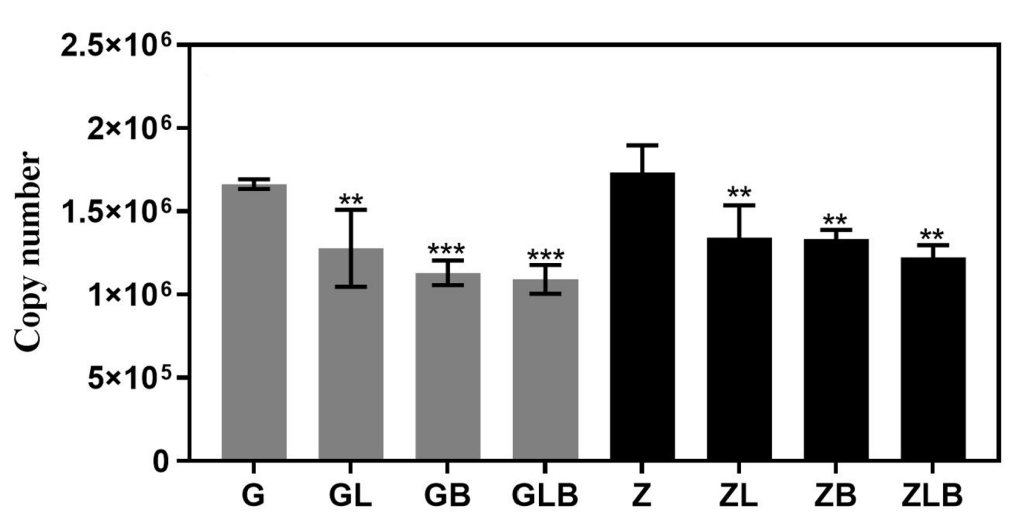


Figure S2. Response biochar and lime amendments on the copy number of *Fusarium* in different trearments of sugarcane rhizosphere

Note: A: Absolute quantitative standard curve; B: copy number of Fusarium. G: no application to GT42 variety; GL: application of 1.5 t ha^-1^ lime to GT42; GB: application of 15 t ha^-1^ biochar to GT42; GLB: combined application of 1.5 t ha^-1^ lime and 15 t ha^-1^ biochar to GT42; Z: no application to ZZ9 variety; ZL: application of 1.5 t ha^-1^ lime to ZZ9; ZB: application of 15 t ha^-1^ of biochar to ZZ9; ZLB: combined application of 1.5 t ha^-1^ lime and 15 t ha^-1^ of biochar to ZZ9. * The t-test showed significant differences (*, p-value < 0.05; * *, p-value < 0.01; * *, p-value < 0.001).
